# Self-reported symptoms in Swedish hairdressers and association with exposure to volatile organic compounds (VOCs), including aldehydes

**DOI:** 10.1186/s12889-023-16446-5

**Published:** 2023-08-18

**Authors:** Niklas Ricklund, Ing-Liss Bryngelsson, Jessika Hagberg

**Affiliations:** 1 Department of Occupational and Environmental Medicine, Örebro University Hospital, Region Örebro County, PO Box 1613, 701 16 Örebro, Sweden; 2 Department of Occupational and Environmental Health, Faculty of Business, Science and Engineering, Örebro University, 70182 Örebro, Sweden

**Keywords:** Indoor air quality, Healthy worker effect, Risk assessment, Hazard index, Hair salon, Airway symptom

## Abstract

**Background:**

Working as a hairdresser involves combined exposure to multiple chemicals in hair treatment products that may induce symptoms in airways and skin.

**Methods:**

In this cross-sectional study, perceived symptoms among Swedish hairdressers at 10 hair salons were surveyed through a questionnaire. Associations with personal exposure to volatile organic compounds (VOCs), including aldehydes, and their corresponding hazard index (HI), based on the estimated risk for non-cancer health effects, were examined. The prevalence of four out of 11 symptoms was compared to available reference datasets from two other studies of office workers and school staff.

**Results:**

All 11 surveyed symptoms were reported among the hairdressers (*n* = 38). For the whole study group, the most prevalent symptoms were *dripping nose* (*n* = 7) and *headache* (*n* = 7), followed by *eczema* (*n* = 6), *stuffed nose* (*n* = 5), *cough* (*n* = 5) and *discomfort with strong odors* (*n* = 5). Significant relationships between exposure and symptoms were scarce. The exception was total VOC (TVOC) exposure adjusted to worked years in the profession; a difference was observed for *any symptom* between hairdressers in the group with 20 + years compared to 0–5 years in the profession (logistic regression, OR 0.03, 95% CI 0.001–0.70). Out of the four symptoms available for comparison, the prevalence of *headache* and *cough* was significantly higher in hairdressers than in controls (OR 5.18, 95% CI 1.86–13.43 and OR 4.68, 95% CI 1.17–16.07, respectively).

**Conclusions:**

Adverse health effects related to occupation was common among the hairdressers, implying a need for exposure control measures in hair salons. Symptoms of *headache* and *cough* were more frequently reported by hairdressers than staff in offices and schools. A healthy worker effect among the hairdressers was indicated in the group with 20 + years compared to 0–5 years in the profession. Significant relationships between measured exposure and symptoms were scarce but gave information about advantages and disadvantages of the different exposure measures. The study design could be improved by increasing the size of the study population, using a better match of reference data and increasing the applicability and representability over time of the measured exposure.

**Supplementary Information:**

The online version contains supplementary material available at 10.1186/s12889-023-16446-5.

## Background

Working as a hairdresser involves combined exposure to multiple chemicals in hair treatment products that may induce various symptoms. In Swedish hairdressers, an increased occupational risk of symptoms has been shown for hand eczema [1], asthma [2] and airway symptoms [3, 4]. An international review of literature between 2014–2019 on exposures among hair and nail salon workers concluded that there was consistent evidence of an increased risk of respiratory effects [5]. Other types of health effects from occupational exposure have also been studied, e.g., reproductive health effects [5, 6], endocrine effects [5] and cancer in different organs [7–13], but conclusions about relationships have so far been inconsistent.

The complexity of chemical exposure in hair salons and variety of symptoms that may be induced in hairdressers necessitate viable and robust methods of risk assessment. For risk assessment of non-cancer health effects from combined exposure to multiple chemicals via indoor air in hair salons, a hazard index (HI) approach was proposed by de Gennaro et al. [14]. The approach concerned volatile organic compounds (VOCs) that are constituents of most hair treatment products. The HI was based on the sum of quotients of measured VOC indoor air concentrations and their corresponding reference values, i.e., concentration below which chronic exposure to a single VOC is unlikely to cause non-cancer health effects. The HI approach was also applied in a recent Swedish study of hairdressers, where an excessive exposure risk was found in four out of 10 hair salons [15]. The HI approach is in line with recommendations in the WHO/IPCS framework concerning a general methodology for risk assessment of combined exposure to multiple chemicals [16] and has been applied in other indoor environments, e.g., homes, schools and offices [17], beauty salons [18], and preschools and primary schools [19, 20].

The main objectives of the present study were to monitor perceived symptoms among a cross-section of Swedish hairdressers and to assess associations with exposure to VOCs, including aldehydes, and their corresponding HI based on the estimated risk for non-cancer health effects.

## Methods

### Study design

A cross-sectional study of the prevalence of self-reported perceived symptoms in hairdressers was conducted in spring 2017 in Örebro County, Sweden. For some of the surveyed symptoms, the prevalence could be compared with reference datasets from two other studies of symptoms in office workers and school staff [21, 22]. Furthermore, associations between the symptoms and personal chemical exposure were assessed. Data on chemical exposure were obtained from measurements conducted in hair salons previously reported by Ricklund et al. [15].

Ethical approval for the study was granted by the Swedish Ethical Review Authority (decision no 2017/414).

### Study group

For the questionnaire survey, a total of 38 hairdressers distributed over 10 hair salons were recruited, with three hairdressers from each hair salon for which chemical exposure data was available.

The inclusion criteria were as follows: working as a professional hairdresser at one of the participating hair salons, which had to have at least three hairdressers available for exposure measurements. The number of participants in the study was based on practical considerations, primarily the plausible participation share of the hair salons.

The recruitment procedure involved identification of an initial number of 44 hair salons in the central town of Örebro. The identified salons were contacted in alphabetical order by telephone during the very same day. Eighteen salons did not respond to the phone call. Ten salons did not agree to participate and were not asked to provide a reason. Three salons could not give an answer on the day but agreed to be contacted again if there were too few participating salons after the first round of recruitment attempts. Three salons agreed to participate but were excluded because they had fewer than three hairdressers available for exposure measurements. Ten salons with at least three hairdressers available for exposure measurements agreed to participate and therefore directly qualified. All included hair salons were well attended, they offered all kinds of common hair treatments and products of internationally established brands and they were not targeted toward any particular customer groups.

### Questionnaire

Symptoms among the hairdressers were self-reported using a questionnaire presented in the Supplementary Information. Included questions concerned demographic data, years in the profession, perceived adverse health effects (mainly related to airway symptoms) and confounding factors, e.g., smoking habits and allergies. The questionnaires were administered personally to the respondents and completed individually without any further instructions.

### Control group

Comparison of self-reported medical symptoms between the study group and a control group was performed. Prevalence data for symptoms *headache, difficulty with concentration, hoarseness* and *cough* were available for a control group (*n* = 319) comprising office workers and school staff obtained previously for indoor climate questionnaire surveys at the Department of Occupational and Environmental Medicine [22]. Another set of reference data on symptoms of *stuffed nose* and *dripping nose* among office workers (*n* = 50), was available from Westerlund [21]. The questionnaires utilized in the two different reference datasets differed from each other and from the questionnaire utilized in the study group, but the compared questions about symptoms were analogous in the different groups. The questionnaires were administered in the same manner for both the controls and the studied hairdressers.

### Chemical exposure

Data on the chemical exposure of the hairdressers were obtained from Ricklund et al. [15]. Measurements of VOCs, including aldehydes, were obtained from personal air sampling in the breathing zone of hairdressers during approximately three hours of work at the hair salons, in conjunction with administration of the questionnaires. Exposure concentrations of individual substances were aggregated to exposure measures expressed as total VOC concentrations (TVOC) and a hazard index (HI). Values of TVOC > median (460 µg/m^3^) and HI > 1 were selected as delimiters between high and low exposure. HI represented the potential risk for non-cancer health effects, as described by De Brouwere et al. [17]. Additional practical considerations regarding HI and its application to the hairdressers, as well as the procedures used for sampling and chemical analysis, are presented elsewhere [15].

### Statistical analysis

Descriptive statistics of the study population were collected, including age, length of employment, different types of hair treatment and health data. Standard parameters such as arithmetic mean (AM), standard deviation (SD), geometric mean (GM) and geometric standard deviation (GSD) were calculated.

Multiple logistic regression was used to analyze whether exposure correlated with perceived symptoms at work, unadjusted or adjusted for years in the profession. Similarly, the relationship between exposure at work and years in the profession was analyzed. Differences between hairdressers and the controls in reference datasets were examined by comparison of the calculated odds ratio (OR) with 95% confidence interval (CI) for overlapping symptoms at work or during spare-time between the groups. Symptoms reported by < 5 hairdressers were only considered in the total sum of symptoms (“*any symptom”*) but were otherwise excluded from the statistical analysis. IBM SPSS Statistics 28.0 was used to perform the statistical analysis. *p* values ≤ 0.05 were considered statistically significant.

## Results

### Medical symptoms

All 38 included hairdressers completed the questionnaire. The characteristics of the study group are presented in Table 1. The results showed that 17 out of the 38 hairdressers (45%) experienced at least one of the 11 surveyed symptoms at work, potentially related to chemical exposure (Table 2). All the 11 surveyed symptoms were reported among the hairdressers. The most commonly reported symptoms at work were *dripping nose* (*n* = 7), *headache* (*n* = 7), *eczema* (*n* = 6), *stuffed nose* (*n* = 5), *cough* (*n* = 5) and *discomfort with strong odors* (*n* = 5).

**Table 1:** Background characteristics of hairdressers (*n* = 38) acquired from the questionnaire survey

	Count	%
Age		
≤ 29	9	24
30 – 39	8	21
40–49	14	37
50 +	7	18
Years in profession		
0–5	8	21
6–20	13	34
21 +	17	45
Working hours per week		
≤ 30	4	11
31 – 40	28	78
41 +	4	11
Gender		
Male	4	11
Female	34	90
Current employment		
Self-employed	28	74
Employee	10	26
Trainee	0	0
Allergy		
No	30	79
Yes	8	21
Smoking habits		
Never	22	61
Former	8	22
Current	6	17

**Table 2:** Results from questionnaire survey of symptoms at work among Swedish hairdressers in total and by years in profession

	Years in profession							
	0–5		6–20		21 +		Total	
	*n*	%	*n*	%	*n*	%	*n*	%
Any symptom								
No	3	37.5	6	46.2	12	70.6	21	55.3
Yes	5	62.5	7	53.8	5	29.4	17	44.7
Watery eyes								
No	6	75.0	12	92.3	17	100	35	92.1
Yes	2	25.0	1	7.7	0	0.0	3	7.9
Dripping nose								
No	6	75.0	10	76.9	15	88.2	31	81.6
Yes	2	25.0	3	23.1	2	11.8	7	18.4
Stuffed nose								
No	7	87.5	9	69.2	17	100	33	86.8
Yes	1	12.5	4	30.8	0	0	5	13.2
Cough								
No	7	87.5	10	76.9	16	94.1	33	86.8
Yes	1	12.5	3	23.1	1	5.9	5	13.2
Hoarseness								
No	8	100	12	92.3	16	94.1	36	94.7
Yes	0	0	1	7.7	1	5.9	2	5.3
Asthma								
No	8	100	12	92.3	16	94.1	36	94.7
Yes	0	0	1	7.7	1	5.9	2	5.3
Headache								
No	7	87.5	9	69.2	15	88.2	31	81.6
Yes	1	12.5	4	30.8	2	11.8	7	18.4
Difficulty with concentration								
No	8	100	12	92.3	17	100	37	97.4
Yes	0	0	1	7.7	0	0	1	2.6
Eczema								
No	5	62.5	12	92.3	15	88.2	32	84.2
Yes	3	37.5	1	7.7	2	11.8	6	15.8
Erythema								
No	4	50.0	13	100	17	100	34	89.5
Yes	4	50.0	0	0	0	0.0	4	10.5
Discomfort with strong odors								
No	5	62.5	12	92.3	16	94.1	33	86.8
Yes	3	37.5	1	7.7	1	5.9	5	13.2

Among the hairdressers with 0–5, 6–20 or 20 + years in the profession, the prevalence of *any symptom at work* was 63, 54 and 29%, respectively. In other words, the prevalence of symptoms was inversely related to the number of accumulated years in the profession, but the relationship was not significant (*p* = 0.06).

Medical symptoms available for comparison between the study group and control group, at work or undefined, showed a significantly higher prevalence of *headache* and *cough* (OR 5.18, 95% CI 1.86–13.43 and OR 4.68, 95% CI 1.17–16.07, respectively) among the hairdressers (Table 3). Regarding *stuffed nose* and *dripping nose,* no significant difference was observed between the study group and control group (OR 0.31, 95% CI 0.08–1.04 and OR 0.62, 95% CI 0.20–1.84, respectively). No comparison was made for *difficulty with concentration* and *hoarseness* because of too few (< 5) reported symptoms among the hairdressers.

**Table 3:** Symptoms among Swedish hairdressers compared to control groups presented as OR (odds ratio). Bold = significant at *p* ≤ 0.05. Results are presented only for symptoms reported by ≥ 5 hairdressers

Symptom	”yes” (%)	OR	95% CI
Headache			
Controls^a^	18 (6)	1	
Hairdressers	7 (18)	**5.18**	**1.86–13.43**
Cough			
Controls^a^	10 (3)	1	
Hairdressers	5 (13)	**4.68**	**1.17–16.07**
Dripping nose			
Controls^b^	15 (31)	1	
Hairdressers	7 (18)	0.62	0.20–1.84
Stuffed nose			
Controls^b^	16 (33)	1	
Hairdressers	5 (13)	0.31	0.08–1.04

^a^Control group of office workers and school staff (*n* = 319) previously utilized for indoor climate questionnaire surveys at Occupational and Environmental Medicine, Örebro University Hospital [22]

^b^Control group of municipal office workers (*n* = 50) [21]

### Significance of exposure for symptoms

Relationships between exposure expressed as HI and symptoms at work among the 30 hairdressers for which exposure data was available showed no statistically significant increased risk of symptoms for exposed hairdressers (Table 4). However, high ORs (> 1) were observed for the symptoms *stuffed nose, cough* (both OR 1.60, 95% CI 0.19–13.24) and *headache* (OR 1.67, 95% CI 0.28–10.09).

**Table 4:** Multiple logistic regression analysis of exposure expressed as HI and TVOC (µg/m^3^) vs. symptoms. Results are presented as unadjusted ORs, with the exception of *any symptom* adjusted for the duration of employment. Bold = significant at *p* ≤ 0.05. Results are presented only for symptoms reported by ≥ 5 hairdressers

		HI	OR	95% CI		TVOC	OR	95% CI
Any symptom		≤ 1.00	1			≤ 460	1	
		> 1.00	0.24	(0.04–1.67)		> 460	0.11	(0.01–1.05)
	*Years in*	*0–5*	1		*Years in*	*0–5*	1	
	*profession*	*6–20*	0.64	(0.04–9.75)	*profession*	*6–20*	0.32	(0.02–4.80)
		*20* +	0.09	(0.01–1.18)		*20* +	**0.03**	**(0.001–0.70)**
Dripping nose		≤ 1.00	1			≤ 460	1	
		> 1.00	0.18	(0.02–1.76)		> 460	0.31	(0.05–1.93)
Stuffed nose		≤ 1.00	1			≤ 460	1	
		> 1.00	1.60	(0.19–13.24)		> 460	3.50	(0.32–38.23)
Headache		≤ 1.00	1			≤ 460	1	
		> 1.00	1.67	(0.28–10.09)		> 460	0.42	(0.07–2.77)
Eczema		≤ 1.00	1			≤ 460	1	
		> 1.00	0.46	(0.04–4.98)		> 460	1.00	(0.12–8.21)
Discomfort with strong odors		≤ 1.00	1			≤ 460	1	
		> 1.00	0.46	(0.04–4.98)		> 460	3.50	(0.32–38.23)
Cough		≤ 1.00	1			≤ 460	1	
		> 1.00	1.60	(0.19–13.24)		> 460	1.00	(0.12–8.21)

Analysis of relationships between exposure expressed as TVOC and symptoms also showed no statistically significant increased risk of symptoms for exposed hairdressers (Table 4), but high ORs (> 1) were observed for the symptoms *stuffed nose* and *discomfort with strong odors* (both OR 3.50, 95% CI 0.32–38.23). However, after adjustment of the TVOC exposure to worked years in the profession, a significant difference was observed for *any symptom* between hairdressers in the group with 0–5 years compared to 20 + years in the profession (logistic regression, OR 0.03, 95% CI 0.001–0.70). This relationship was not detected between HI and symptoms. Neither was a difference observed for *any symptom* adjusted to TVOC exposure between the groups with 0–5 and 6–20 years in the profession (OR 0.32, 95% CI 0.02–4.80) nor between the groups with 6–20 and 20 + years in the profession (OR 0.11, 95% CI 0.01–1.09).

Considering solely exposure, it did not significantly differ between the groups with 0–5 compared to 6–20 and 20 + years in the profession. Regression analysis gave the following results for TVOC: OR 1.0, 95% CI 0.112–8.947; OR 0.44, 95% CI 0.056–3.508, respectively. Corresponding results for HI were OR 6.0, 95% CI 0.48–73.34; and OR 2.0, 95% CI 0.17–22.95.

## Discussion

### Symptoms among hairdressers

The prevalence of symptoms at work reported by the hairdressers (*any symptom* 45%, all 11 symptoms reported at least once) showed that adverse health effects related to their occupation were common and more than twice as common as symptoms related to indoor environments reported by the general population in Sweden [23]. Evaluation of symptom prevalence among hairdressers compared to available reference datasets for *headache, cough*, *stuffed nose* and *dripping nose* showed a significant increased risk of *headache* and *cough* among the hairdressers (Table 3), suggesting that these symptoms were more frequent among hairdressers compared to staff in other indoor environments of offices and schools. However, the evaluation was limited by the overlap of questions in the questionnaires utilized in the two different control groups and symptoms reported by too few hairdressers, i.e., fewer than 5 hairdressers reported *difficulty with concentration* and *hoarseness*. A better match of reference data with the questionnaire data of the study group could possibly have shown additional differences in symptoms between the hairdressers compared to controls.

The reported risk in the literature concerning different forms of *cough* among hairdressers compared to non-hairdressing controls varies from no increased risk [24–26] to a significantly increased risk, e.g., ranging from two- [27] or three-fold [3, 28] lower than that in the present study to three-fold higher than in the present study [29]. Specifically, in a French study, no increased risk was found of *cough during morning or day or night* in hairdressers compared to office worker apprentices during their first year and at a five-year follow-up [24]. Neither was there an increased risk of *cough during* > *14 days* reported for hairdressers compared to office workers in a Norwegian study [25]. Similarly, no increased risk of *cough at work* was observed in hairdressers compared to office workers in a study from Greece [26]. On the other hand, an increased risk of *dry cough* among hairdressers compared to the general population was demonstrated in Sweden (incidence rate ratio (IRR) 1.5, 95% CI 1.2–1.9) [3] and an increased risk of *cough with phlegm* and *dyspnoea with cough* among hairdressers compared to saleswomen was shown in Finland (OR 1.4, 95% CI 1.1–1.9 and OR 1.6, 95% CI 1.0–2.7, respectively) [28]. Furthermore, the risk of *dry cough* was higher among hairdressers compared to office workers in a recent study from Iran (OR 2.18, 95% CI 1.26–3.77) [27] and the risk of *work-related cough* in hairdressers compared to non-hairdressing controls was reported to be higher in a study from the UK (OR 13.2, 95% CI 1.3–131.5) [29]. However, interpretation of the underlying reasons for the variation in risk quotients between studies is precarious due to multifactorial differences concerning study subjects, controls and methods.

No data on *headache, difficulty with concentration* or *hoarseness* among hairdressers compared to controls have been reported in the literature, but corresponding data concerning symptoms in the nose and other symptoms related to chemical exposure via air, i.e., airways and eyes, skin excluded, are available. Excessive risks of *wheezing* (OR 2.1, 95% CI 1.6–2.7) and *nasal blockage* (OR 3.0–5.4, 95% CI 1.9–7.6) in Swedish active hairdressers compared to the general population were found by Brisman et al. [3], an increased risk of *rhinitis* (OR 1.59, 95% CI 1.30–1.98) but not other symptoms of *wheezing* and *asthma* was reported for Danish hairdressers compared to the general population [30], and increased risks of *rhinitis* (OR 1.7, OR 95% CI 1.3–2.3), *rhinitis with eye symptoms* (OR 1.9, 95% CI 1.4–2.6), *dyspnoea* (OR 1.5, 95% CI 1.0–2.2) and *chronic bronchitis* (OR 4.8, 95% CI 2.2 to 10.1) but not *allergic rhinitis* or *asthma* or *laryngitis* were found for Finnish hairdressers [28]. Higher risks of *dyspnea at work*, *irritation of eyes at work* and *irritation in the throat at work* were observed in Greek hairdressers compared to office workers (*p* values 0.026, 0.001 and 0.009, respectively) [26]. However, in the same study, corresponding data for *sputum production at work*, *wheezing at work* and *irritation in the nose at work* did not show an increased risk [26]. No excessive risks of *wheeze*, *chest tightness* or *asthma* among hairdressers compared to non-hairdressing controls were found by Bradshaw et al. [29] and no increased risk of *wheezing* and other symptoms, including *dyspnoea* (or deterioration of respiratory functions), was detected in French hairdresser apprentices compared to office worker apprentices [24]. However, in contrast to the findings of the Danish and Finnish studies, Albin et al. [2] showed an increased risk of *asthma* (IRR 1.3, 95% CI 1.0–1.6) in Swedish active hairdressers compared to the general population. Likewise, Ghosh et al. [31] detected a higher risk of *adult onset asthma* (OR 1.88, 95% CI 1.24 to 2.85) among British hairdressers compared to unexposed individuals in low-risk jobs.

In all the abovementioned studies and for all the symptoms for which an increased risk has been reported among hairdressers compared to non-hairdressing controls, the calculated risk expressed as OR or IRR, when available, was statistically significant (with 95% CI) but moderate at 1.3–2.5, with the exception of *nasal blockage* with higher ORs between 3.0–5.4 reported by Brisman et al. [3], *chronic bronchitis* with OR of 4.8 reported by Leino et al. [28] and *work-related cough* with OR of 13.2 reported by Bradshaw et al. [29]. Nevertheless, findings of different studies on the risk of symptoms among hairdressers compared to non-hairdressing controls are not unequivocal, although there are strong consistencies concerning, e.g., (non-allergic) rhinitis. Some of the discrepancies in results may be due to methodological considerations, which may have affected the estimated risk, hampering comparisons between studies. It is also possible that different trends in chemical composition of hair treatment products and working procedures over time and between geographical regions may have contributed to different results between studies. Furthermore, adjustments of data have been performed for different factors between studies, including smoking, atopy and age [2, 25, 28], geographical region [2, 30], social class at birth [31], education level [30] and worked years [29]. In the present study, the data were only adjusted for worked years – the study population was considered too small for additional adjustments.

### Symptoms in relation to exposure

The prevalence of symptoms among hairdressers and increased risk of certain symptoms (*headache* and *cough*) among hairdressers compared to controls (Table 3) may have been due to inhalation exposure of chemicals from hair treatment products, suggesting a need for exposure control measures in hair salons. Symptoms for which no statistically significant differences to controls were observed, i.e., *stuffed nose* and *dripping nose*, could be due to consequential exposure in the reference environments (e.g., airborne dust or chemicals, pet allergens), lack of causality between chemical exposure and symptoms among the hairdressers or the small size of study group. None of these possible explanations could be verified or rejected within the present study.

Exposure of the hairdressers expressed as TVOC > median or HI > 1 did not show a statistically significant increased risk for six out of the 11 symptoms included in the analysis (Table 4). However, for exposure expressed as TVOC > median, non-significant high ORs (3.50) were observed for two symptoms, i.e., *stuffed nose* and *discomfort with strong odors*. Similarly, exposure expressed as HI > 1 generated non-significant results but high ORs (1.60–1.67) for three symptoms – *stuffed nose, cough* and *headache*. Exposure expressed as both TVOC and HI showed a high OR for *stuffed nose*. The other two symptoms with high ORs for exposure expressed as HI, i.e., *cough* and *headache,* which were statistically significantly more common among the hairdressers compared to the controls, could imply a higher sensitivity of this measure for prediction of risk for certain symptoms. A high OR for *discomfort with strong odors* was only observed for TVOC. This may suggest that TVOC, to a larger extent than HI, was proportional to the volumetric usage of hair treatment products in the hair salons, and therefore also the aggregated strength of odor. Thus, TVOC exposure could reflect the working practice of the hairdressers. It is also possible that another delimiter, apart from the median concentration of TVOC between hairdressers with low and high exposure, could increase the sensitivity of this exposure measure for detecting symptoms. This would have been feasible to test with a larger population sample. However, the non-significant results concerning relationships between exposure and symptoms prevent definite conclusions.

The lack of statistically significant associations between symptoms and exposure measures may be explained by similar reasoning, i.e., possible lack of causality between chemical exposure and symptoms or too small a study group. Furthermore, limitations of the exposure measures may have interfered. It is plausible that the measured exposure was affected by a lack of representativeness to reflect exposure over time to develop symptoms. Concerning the exposure expressed as TVOC, its potential casualty with symptom prevalence may have been limited by the fact that it only represented a sum of concentrations and did not consider potential health risks. High values of TVOC do not by definition indicate high risk, in contrast to HI values. On the other hand, HI may have been affected by limitations in the input data, such as the lack of available reference values for the included chemicals, as well as the potential presence of chemicals causing health effects that were not included in the sampling method [15]. Examples of such chemicals are hydrogen peroxide in permanent wave solution, bleaching powder and dyes, thioglycolic acid and ammonia in permanent wave solution, persulfates in bleaching powder, and toluenediamine and phenylenediamine compounds in dyes. In other words, increasing the input of data in the HI calculation could increase the applicability of the exposure measure and help clarify association patterns between exposure and symptoms.

Low ORs, also statistically non-significant, were observed for the two measures of exposure and risk of the symptoms *eczema*, *dripping nose* and *asthma* (Table 4). The connection between exposure to hair treatment products and *asthma* among Swedish hairdressers was also studied by Albin et al. [2]. Their results suggested that asthma could be related to high usage of bleaching products (≥ 8 compared to 0–1 treatments/week) and hairspray (≥ 51 compared to 0–30 treatments/week), although the relationships were not statistically significant. Calculated IRR values were 1.5 (95% CI 0.7–3.0) and 1.4 (95% CI 0.8–2.4), respectively. A connection between bleaching agents and asthma was also shown in an Italian study of 47 hairdressers with suspected occupational asthma (and other airway symptoms) [32]. Twenty four of the study subjects were diagnosed with the disease after a specific inhalation challenge, indicating that persulfate salts in bleaching products, permanent hair dyes and latex were the causal agents in 11, two and one cases, respectively.

Causality between hairspray, bleaching agents and airway symptoms other than asthma has also been described in the literature. Self-reported data by hairdressers showed that hairspray and bleaching powder, among commonly utilized hair treatment products, were the most strongly provoking of various airway symptoms [33]. In a study of Swedish hairdressers with and without nasal symptoms, it was shown that usage of hairspray, among several inventoried exposure factors, was significantly higher in the group with symptoms [4]. Concerning the exposure of hairdressers to persulfates, data are readily available from provocation studies showing an increase of airway symptoms, particularly in symptomatic hairdressers [4, 32, 34–36], although the increase in some cases was small [37]. In addition, a response of different biomarkers has been demonstrated [34, 35, 38–40]. In a recent review of effects in the airways of hairdressers following usage of persulfate salts, it was concluded that this group of substances was likely the main cause of occupational asthma and rhinitis [41].

Relationships between other exposure factors related to inhalation air and symptoms in hairdressers have been reported less commonly. In a study of 33 Hebron non-smoking hairdressers, exposure to ammonia was found to not be associated with inflammatory markers in sputum, self-reported respiratory symptoms or lung function [42]. However, exposure measurements revealed episodes of hazardous exposure compared to occupational limit values and several of the examined effects were significantly more pronounced among the hairdressers compared to controls. Ammonia was not included in the exposure measurements of the present study. In another study of 36 non-smoking workers from different beauty salons in Tehran, exposure via indoor air to benzene, toluene, ethylbenzene and xylenes (BTEX) was associated with *irritation in eyes, nose, throat,* and *lung*, and in one case for toluene, *menstrual disorders* were observed [18]. In the same study, urinary concentrations of the analyzed chemicals were higher post-shift compared to pre-shift and controls. In addition, HI for BTEX exposure was calculated but was much lower than 1 in all cases. The authors argued that the measured concentrations could be underestimates since they represented background and not personal exposure. However, since only a handful of substances were analyzed, the total exposure likely involved many more chemicals that could potentially make substantial contributions to summed hazard ratios. For comparison, BTEX compounds in the present study were only identified in a few samples and at relatively low concentrations, e.g., benzene and ethylbenzene were not identified in any samples, toluene was found in six samples (in range 14–31 µg/m^3^) and xylene was found in one sample (at 3 µg/m^3^), while the total number of identified chemicals was more than 90 [15].

The effects of physical features of the indoor environment of hair salons and workload on the prevalence of symptoms in hairdressers have also been examined in different studies. Installation of local exhaust ventilation was suggested to have led to improvement of respiratory symptoms over time among hairdressers in Norway [43]. In a study from Greece, hair salons with a larger working area and characteristics of ventilation (presence of windows) were associated with better lung function among the studied hairdressers [26]. However, the studied health effects were not associated with estimated work intensity (self-reported number of specific treatments/week), which, according to the authors, could therefore be an insufficient measure of exposure. This finding is in line with the results of the present study, where qualitatively assessed co-variations between hair treatments and patterns of chemical exposure were inconsistent.

For symptoms in the skin, the relevance of chemical exposure via inhalation air was suggested to be small in the present study. Rather, symptoms in the skin are typically related to wet work and direct contact with chemicals in hair treatment products. Therefore, the applied exposure measures (HI and TVOC) might be insufficient for prediction of such symptoms. Nevertheless, hand eczema among hairdressers is common. In a study population of Swedish hairdressers between the years 1970–1995, the occurrence of self-reported hand eczema expressed as one-year prevalence was estimated as 18%, compared to 12.1% for controls [1]. IRR between the groups was 2.5 (95% CI 2.2 to 2.8) and was higher for young hairdressers < 25 years of age (IRR 3.1, 95% CI 2.6 to 3.5).

### Healthy worker effect

The finding of an inverse relationship between prevalence of *any symptom* and number of accumulated years in the profession (0–5, 6–20 or 20 +) for the studied hairdressers may have a couple of explanations. The variation between groups may reflect differences in predisposition for developing symptoms among the hairdressers. Alternatively, the differences could correspond to different group patterns of working procedures promoting exposure. If so, TVOC exposure could serve as an indicator of working practices promoting exposure. However, exposure represented by TVOC did not differ significantly between the groups of hairdressers with 0–5 and 6–20 or 20 + years in the profession (OR 1.0, 95% CI 0.112–8.947; OR 0.44, 95% CI 0.056–3.508, respectively). On the other hand, TVOC exposure adjusted to years worked in the profession showed a significant difference for *any symptom* between hairdressers at the beginning of their career compared to the most experienced hairdressers (20 + compared to 0–5 years in the profession; OR 0.03, 95% CI 0.001–0.70). Altogether, these findings may be due to a combined effect of working practice and predisposition for developing symptoms, indicating a so-called healthy worker effect in the group of hairdressers with 20 + years in profession.

Patterns of healthy worker effects in hairdressers are not consistent in the literature. In a Norwegian cross-sectional study, a healthy worker effect was proposed for eczema in hairdressers aged > 40 years, although such an effect was not observed for airway symptoms [25]. In a later prospective study from Norway in which airway symptoms and biomarkers were studied after installation of local exhaust ventilation, hairdressers in the study population remaining in the profession over the time period 1995 to 1999, i.e., only 60%, were suggested to be a highly selected and healthy group of workers [43]. A healthy worker effect in relation to asthma has been suggested for Danish hairdressers [44] as well as Danish hairdresser apprentices [30]. However, in the latter study, the prevalence of rhinitis was higher in third year apprentices than in first year apprentices. A similar result was found in an Italian prospective study of hairdressers during the years 2006–2016, which showed that the prevalence of irritant skin and upper respiratory symptoms increased significantly over the study period [45]. In other words, a healthy worker effect did not seem to be apparent. Likewise, more respiratory symptoms were observed at follow-up in a five-year prospective study of Palestinian hairdressers, and working for more years was associated with lung function decline [46]. In a recent Iranian cross-sectional study of 140 hairdressers, increased duration of work in the profession was related to an increased risk of respiratory symptoms and decreased lung function [27]. Nevertheless, among hairdressers with the longest duration in work (> 15 years), a plateau effect was observed, likely due to a healthy worker effect, according to the authors. It is noteworthy that the plateau effect for irritative responses among the hairdressers appeared after > 15 years of work. This is similar to the exposure duration in the present study, where a similar effect was suggested for hairdressers with 20 + years in profession. Furthermore, both these exposure periods are longer than those used in the aforementioned prospective studies, which did not detect a healthy worker effect. Therefore, for at least some symptoms, the duration of the prospective studies might have been too short to observe the effect.

## Conclusions

Various adverse health effects related to occupation were commonly reported by hairdressers. Thus, there is a need for exposure control measures in hair salons to decrease the risk of symptoms among hairdressers. Symptoms of *headache* and *cough* were more frequently reported by hairdressers than staff in offices and schools. Additional differences in symptoms between hairdressers compared to controls could not be ruled out due to limitations of the reference data. A healthy worker effect among the hairdressers was indicated in the group with 20 + years compared to that with 0–5 years in the profession. Significant relationships between measured exposure and symptoms were scarce. Nevertheless, information was gained concerning the advantages and disadvantages of applying different exposure measures, i.e., HI vs. TVOC. The study design could be improved by increasing the size of the study population, using a better match of reference data, and increasing the applicability and representability over time of the measured exposure.

### Supplementary Information

**Additional file 1. **Health survey for hairdressers.

## Data Availability

The data underlying this article are available in the article and in its online Supplementary Information.

## Abbreviations

HI: Hazard index
TVOC: Total volatile organic compound
BTEX: Benzene, toluene, ethylbenzene and xylenes
