# Supplementary material for: Self-reported symptoms in Swedish hairdressers and association with exposure to volatile organic compounds (VOCs), including aldehydes
Source: BMC Public Health. 2023 Aug 18;23:1576. doi: 10.1186/s12889-023-16446-5 (PMC10436395; doi:10.1186/s12889-023-16446-5)
Supplement: Supplementary file 1 — Additional file 1. Health survey for hairdressers. [file 12889_2023_16446_MOESM1_ESM.docx]

## Supplementary Information

**Health survey for hairdressers**

Serial number:

Year of birth: ______________________

Gender: Male

Female

Current employment: Self-employed

Employee

Trainee

Years in profession: 0-5 years

6-15 years

16-20 years

21-30 years

31-45 years

Approximate number of working hours per week: ______________________

Mark the symptoms below that you perceive and when the symptoms appear, i.e., at work or at spare-time or both. Specify for how long you have experienced each symptom and the where on your body skin symptoms, if any, occur.

| Type of symptom | Symptom perceived at work | Symptom perceived during spare-time | How long symptoms have been perceived | Place on body (symptoms of the skin) |
| --- | --- | --- | --- | --- |
| Watery eyes |  |  |  |  |
| Dripping nose |  |  |  |  |
| Stuffed nose |  |  |  |  |
| Cough |  |  |  |  |
| Hoarseness |  |  |  |  |
| Asthma |  |  |  |  |
| Headache |  |  |  |  |
| Difficulty with concentration |  |  |  |  |
| Eczema (specify place on your body) |  |  |  |  |
| Erythema (specify place on your body) |  |  |  |  |
| Discomfort with strong odors (e.g. perfumes) |  |  |  |  |

Specify other work-related symptoms, if any: _______________________________________

__________________________________________________________________________________

Specify symptoms, if any, that improve during weekends or vacations:_________________

__________________________________________________________________________________

Do you have allergies: Yes

No

If yes, specify type of allergy:______________________________________________________

If yes, mark if the allergy has been diagnosed by health care: Yes

No

Do you have pets? Yes

No

If yes, specify type of pet(s):_______________________________________________________

Have you experienced stress at work? Often

Sometimes

Seldom

Never

Have you experienced stress at home? Often

Sometimes

Seldom

Never

Do you exercise regularly? Yes (at least twice a week or 1.5 hours/week)

No

Are you a smoker? Yes No

Previous smoker? Yes No

One-time smoker? Yes No

If you are a smoker, specify number of cigarettes/day: ___________________________________________

If you are a previous smoker, specify when you quit: ___________________________________________
